# Evaluation of Real-Life Chemoimmunotherapy Combination in Patients with Metastatic Small Cell Lung Carcinoma (SCLC): A Multicentric Case–Control Study

**DOI:** 10.3390/cancers15184593

**Published:** 2023-09-15

**Authors:** Rémy Ezzedine, Anthony Canellas, Charles Naltet, Marie Wislez, Reza Azarian, Andrei Seferian, Etienne Giroux Leprieur

**Affiliations:** 1 Department of Respiratory Diseases and Thoracic Oncology, APHP—Hôpital Ambroise Paré, 92104 Boulogne-Billancourt, France; remy.ezzedine@aphp.fr; 2 Department of Respiratory Diseases and Thoracic Oncology, APHP—Hôpital Tenon, 75020 Paris, France; anthony.canellas@aphp.fr; 3 Department of Respiratory Diseases, Hôpital Paris Saint Joseph, 75014 Paris, France; cnaltet@ghpsj.fr; 4 Thoracic Oncology Unit, Pulmonology Department, APHP—Hôpital Cochin, Université Paris Cité, 75006 Paris, France; marie.wislez@aphp.fr; 5 Department of Respiratory Diseases, Hôpital Mignot, 78150 Le Chesnay, France; razarian@ght78sud.fr; 6 Department of Respiratory Diseases, APHP—Hôpital Bicêtre, 94270 Le Kremlin-Bicêtre, France; andrei.seferian@aphp.fr

**Keywords:** small-cell lung cancer, atezolizumab, brain metastases, liver metastases, elderly

## Abstract

**Simple Summary:**

Chemoimmunotherapy (CT-IO) is the standard first-line treatment of advanced small cell lung cancer (SCLC). However, very limited efficacy data from real-life use of this combination are available. Moreover, patients included in pivotal phase III trials were highly selected. We conducted a retrospective multicentric (six academic centers) case–control study (n = 153), comparing two cohorts of patients who received first-line treatment, one treated with chemotherapy alone (between January 2017 and December 2018) and one with CT-IO (between January 2019 and December 2020), for an advanced SCLC. CT-IO confirmed its superiority compared to chemo alone. Interestingly, we showed that CT-IO was efficient in patients with brain and liver metastases. However, patients ≥70 years old and with a PS ≥2 did not benefit from CT-IO. This real-life study in one of the largest published to our knowledge on first-line CT-IO for advanced SCLC, with a large focus on specific populations (brain mets, elderly, and impaired PS).

**Abstract:**

The current first-line standard treatment for advanced small cell lung cancer (SCLC) is a combination of chemotherapy and immunotherapy. However, few efficacy data are available in a real-life settings, including frail patients. The aim of this study is to describe the real-life efficacy of chemoimmunotherapy in an unselected SCLC population. We conducted a retrospective multicenter study, which compared two cohorts of patients with treatment-naive metastatic SCLC treated in six academic centers in the Greater Paris area. Cohort 1 included patients treated with chemotherapy between January 2017 and December 2018, and cohort 2 included patients treated with chemoimmunotherapy between January 2019 and December 2020. A total of 153 consecutive patients were included (cohort 1: n = 96; cohort 2: n = 57). Clinical characteristics were similar between the two cohorts. Overall survival (OS) was statistically higher in cohort 2 (median survival 15.47 months) than in cohort 1 (median survival 9.5 months) (p = 0.0001). OS for patients with a performance status ≥2 and for patients ≥70 years old was not statistically different between the two cohorts. Chemoimmunotherapy efficacy was better compared to chemotherapy alone in case of brain or liver metastases. In conclusion, the combination of chemoimmunotherapy in metastatic SCLC appears to provide a real-life OS benefit. Dedicated clinical trials are needed to test this strategy in patients with impaired performance status or advanced age.

## 1. Introduction

Lung cancer is the leading cause of cancer deaths worldwide. Small cell lung histological subtype represents about 15% of lung cancers [1]. Its prognosis remains very poor, with a median survival of 2 to 4 months without treatment and about 10 months with chemotherapy alone [2]. Approximately 50% of small cell lung cancers are diagnosed at the metastatic stage, which partly explains the low 5-year survival rate (4–7%) [3]. For more than 30 years, the standard treatment in the metastatic phase was based on a doublet of platinum-based chemotherapy (CT) [4,5]. However, recent studies have changed this paradigm, making chemoimmunotherapy (CT-IO) the new standard of care in the first-line treatment of patients with metastatic SCLC. Program death ligand-1 (PD-L1), present on the surface of tumor cells, provides a link between the tumor cell and the T lymphocyte, and this link represents a brake on the immune response. Monoclonal anti-PDL1 antibodies (atezolizumab and durvalumab) are able to restore the immune response and ensure the proliferation and recruitment of T cells in the tumor microenvironment [6]. In combination with platinum-based chemotherapy and etoposide, they improved the overall survival (OS) and progression-free survival (PFS) of patients with metastatic SCLC [7,8], changing the previously unchallenged standard of care [9]. In the Impower-133 study, the median OS in the atezolizumab arm was 12.3 months vs. 10.9 months in the placebo arm [7]. Similar results were observed in the CASPIAN study with the use of durvalumab (13 months median OS in the durvalumab arm vs. 10.3 months in the placebo arm) [8]. To date, very few multicentric real-life data are available to validate these results from the phase III clinical trials and are often in small population samples [10,11,12,13]. The objective of this study is to evaluate the efficacy of chemoimmunotherapy as first-line therapy in a real-life cohort of patients compared to a control cohort treated with CT alone. We have also collected data from second-line treatment efficacy in both cohorts, and included populations of special interest, i.e., either elderly patients or impaired general condition.

## 2. Patients and Methods

### 2.1. Patients

All consecutive patients with histologically or cytologically confirmed metastatic SCLC, who were treatment-naive, treated with CT alone (between 1 January 2017 and 31 December 2018) or with CT-IO (between 1 January 2019 and 31 December 2020) in six French academic centers were collected.

In both cohorts, patients had to be older than 18 years, have a measurable disease according to the Response Evaluation Criteria in Solid Tumors (RECIST) version 1.1, and have received at least one cycle of treatment. In both cohorts, patients with corticosteroid therapy greater than 20 mg per day and patients with history of autoimmune disease were excluded. Patients with stable intracranial metastatic lesions were eligible for inclusion.

Patients in cohort 1 (CT alone) received platinum-based chemotherapy (intravenous administration of cisplatin (75 mg/m^2^) or carboplatin (AUC 5)) in combination with etoposide (intravenous administration of 100 mg/m^2^ on D1, D2, and D3) every 21 days for a total of 4 to 6 cycles.

Patients in cohort 2 received chemotherapy with carboplatin (AUC 5), etoposide (intravenous administration of 100 mg/m^2^ on D1, D2, and D3) and atezolizumab (intravenous administration of 1200 mg on D1) every 21 days for a total of 4 cycles, followed by maintenance atezolizumab (intravenous administration of 1200 mg every 21 days).

Prophylactic brain irradiation was used in both cohorts at the discretion of the patient’s referring physician. 

The initial assessment included a thoracic–abdominal–pelvic CT scan and brain imaging (CT or brain MRI). Tumor evaluation was performed every 2 or 3 cycles, depending on the center, using thoracic–abdominal–pelvic CT scan and brain imaging (CT or brain MRI).

### 2.2. Statistical Analysis

The primary endpoints were OS (time from diagnosis to death from any cause) and PFS (time from diagnosis to progression according to RECIST 1.1, or death from any cause) in both cohorts.

Secondary endpoints were objective response rate and PFS with second-line therapy (time between progression at first-line and progression at second-line according to RECIST 1.1 criteria, or death from any cause).

We also retrospectively collected adverse events according to the National Cancer Institute Common Terminology Criteria for Adverse Events (CTCAE) version 4.0 to evaluate the real-life safety of chemoimmunotherapy in this setting of metastatic SCLC in first-line therapy.

Kaplan–Meier methodology was used to estimate the probability of OS and PFS. OS and PFS results were compared using the log-rank test. Objective response rates were compared using a Fisher test. A comparison of the clinical data was performed using a Chi-2 test or Fisher test for variables with n < 5. The cut-off date was 1 April 2022. 

## 3. Results

A total of 153 consecutive patients were included (cohort 1, n = 96; cohort 2, n = 57). The characteristics of the patients in the two cohorts were comparable (Table 1). The patients were mainly male (65% in cohort 1 and 54% in cohort 2), with an average age of 65 years and the vast majority being smokers. Twenty-seven percent of patients in cohort 1 and 48.9% in cohort 2 were 70 years old or older. There were 24.3% of patients with an impaired general condition (PS > 1) in cohort 1, and 10.6% in cohort 2 (p = 0.21). There was no significant difference between the two groups regarding the presence of brain (35% vs. 25%) or liver (45% vs. 44%) metastases. Considering the platinum drug, all the patients in cohort 2 received carboplatin vs. 70% in cohort 1. All the patients received at least one cycle of treatment, and up to 6 cycles (cohort 1) or 4 cycles (cohort 2) of chemotherapy. 

### 3.1. OS and PFS

Median follow-up was 11.9 months. At the time of analysis, 92 patients (95.8%) in cohort 1 and 36 patients (63.2%) in cohort 2 were deceased.

**Figure 1:**
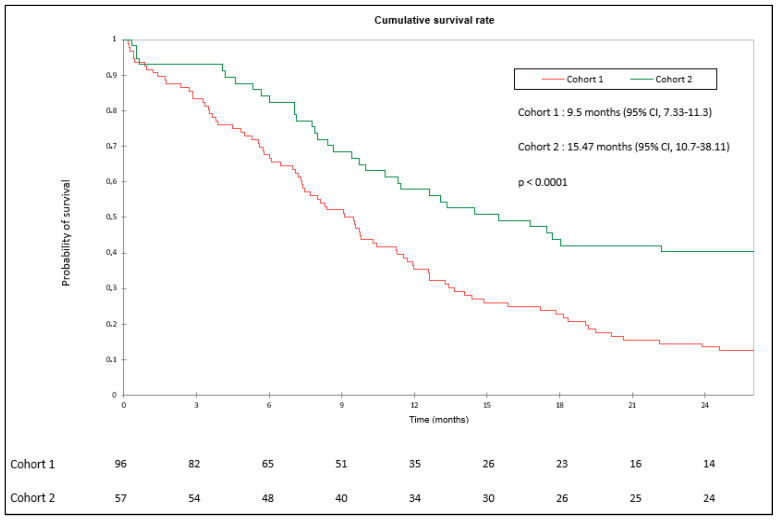
Overall Survival in cohort 1 (chemotherapy) and cohort 2 (chemoimmunotherapy).

OS was statistically higher in cohort 2 (median 15.47 months) than in cohort 1 (median 9.5 months) (*p* < 0.0001). OS in cohorts 1 and 2 were 34.3% and 57.9% at 1 year, respectively. Survival at 2 years in the different cohorts was 14.5% and 42.1%, respectively (Figure 1).

**Figure 2:**
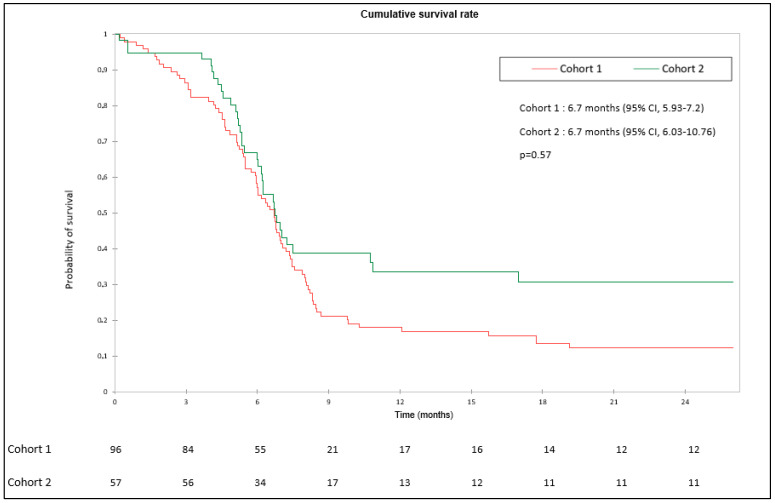
Progression-free survival in cohort 1 (chemotherapy) and cohort 2 (chemoimmunotherapy).

At the time of analysis, 92 patients (95.8%) in cohort 1 and 47 patients (82.4%) in cohort 2 had tumor progression or died. PFS in cohort 2 (median 6.73 months) was not statistically different from that of cohort 1 (median 6.7 months) (*p* = 0.57). PFS in cohorts 1 and 2 were 17.7% and 22.8% at 1 year, respectively. PFS at 2 years in the different cohorts was 12.5% and 19.3%, respectively (Figure 2).

### 3.2. Objective Response Rate

The objective response rate in the cohort 1 was 61.2% and 81% in cohort 2 (*p* = 0.09) (Table 2). The complete response rate in cohorts 1 and 2 were 6.2% and 11%, respectively. Progression rate was also higher in the cohort 1 (26% vs. 8.8%, *p* = 0.043).

### 3.3. Efficacy of 2nd Line Treatment

Forty-nine patients (51%) in cohort 1 and 30 patients (52.6%) in cohort 2 received a second-line treatment at progression. PFS with the second-line treatment was not statistically significant between cohorts 1 and 2 (3.6 months and 2.2 months, respectively) (*p* = 0.23) (Supplementary Figure S1). In cohort 2, PFS was statistically similar in patients who received topotecan, lurbinectedin, or another treatment (*p* = 0.09) (Supplementary Figure S2).

### 3.4. Population of Interest

#### 3.4.1. Brain Metastases

Thirty-five per cent (*n* = 34) in cohort 1 and 25% (*n* = 14) in cohort 2 had brain metastases at diagnosis. The median OS in cohort 1 without brain metastases was 9.7 months vs. 7.8 months with brain metastases (*p* = 0.406). In cohort 2, the median survival without brain metastases was 15.4 months vs. 15.5 months with brain metastases (*p* = 0.62). Patients with brain metastases had a significantly better OS under chemoimmunotherapy vs. chemotherapy alone (15.5 vs. 7.8 months, *p* = 0.011). In patients without brain metastases, OS was also longer with chemoimmunotherapy vs. chemotherapy alone (15.4 vs. 9.7 months, *p* = 0.0002) (Supplementary Figure S3).

#### 3.4.2. Liver Metastases

Forty-five per cent (*n* = 43) in cohort 1 and 44% (*n* = 25) in cohort 2 had liver metastases at diagnosis. The median OS in cohort 1 without liver metastases was 10.3 months vs. 7.7 months without liver metastases (*p* = 0.054). In cohort 2, the median survival without liver metastases was 15.4 months vs. 11.4 months with liver metastases (*p* = 0.33). Patients with liver metastases had a better OS under chemoimmunotherapy (11.4 vs. 7.7 months, *p* = 0.002). In patients without liver metastases, OS was also longer with chemoimmunotherapy (15.4 vs. 10.3 months, *p* = 0.001) (Supplementary Figure S4).

#### 3.4.3. Poor Performance Status

Seventy-six per cent (*n* = 73) in cohort 1 and 90% (*n* = 51) in the cohort 2 were PS 0–1. The median OS in cohort 1 with PS 0–1 was 9.8 months vs. 4.5 months if PS was 2–3 (*p* = 0.018). In cohort 2, the median OS with PS 0–1 was 16.8 months vs. 8.9 months if PS was 2–3 (*p* = 0.329). Patients with PS 0–1 had a better OS under chemoimmunotherapy (16.8 vs. 9.8 months, *p* = 0.001) (Supplementary Figure S5A).

Patients with a PS of 2 or higher did not appear to benefit from chemoimmunotherapy treatment compared to chemotherapy alone: 4.5 months vs. 8.9 (*p* = 0.092) (Supplementary Figure S5B).

#### 3.4.4. Elderly Patients

Twenty-eight per cent (*n* = 27) in cohort 1 and 44% (*n* = 25) in cohort 2 were more than 70 years old. In the cohort 1, the median OS was 10.9 months (≥70 years old) vs. 8.4 months (<70 years old) (*p* = 0.189). In cohort 2, the median survival was 9.4 months (≥70 years old) vs. 24.3 months (<70 years old) (*p* = 0.031). For elderly patients (>70 years old), there was no significant difference in OS in cohort 1 vs. cohort 2 (*p* = 0.43). In patients < 70 years old, OS was significantly better in cohort 2 (*p* = 0.0001) (Supplementary Figure S6).

### 3.5. Safety

A total of 94 patients (97.9%) in cohort 1 and 55 patients (96.5%) in cohort 2 experienced treatment-related adverse events (Table 3).

Endocrinopathy-type adverse events were noted in cohort 2, including four cases of thyroiditis, one case of diabetes, one case of hypophysitis, one case of adrenal insufficiency, and two cases of pancreatitis. There were three cases of interstitial lung disease. There were no treatment-related deaths in cohort 2.

## 4. Discussion

Our study confirms, in a large multicentric real-life cohort, that the combination of atezolizumab with platinum-based CT significantly improved OS compared to a control group of patients treated with CT alone. OS in our study (15.5 months) was comparable to OS in the IMpower—133 trial (12.3 months) that tested atezolizumab in association with chemotherapy in selected patients [7]. Importantly, our study included a large proportion of patients with impaired PS, advanced age, or brain metastases, who were excluded from pivotal phase III trials. In our study, we did not find any benefit of CT-IO combination for patients ≥ 70 years-old or with PS > 1, whereas we found an OS benefit in patients with brain or liver metastases.

A few studies have looked at the efficacy of immunotherapy in elderly patients with lung cancer, mainly NSCLC [14,15], and the results suggested a benefit for these patients. As an example, in the real-world French study of the nivolumab efficacy on NSCLC (*n* = 907), the age at initiation of nivolumab did not impair OS [16]. A meta-analysis of clinical trials with immunotherapy in monotherapy in NSCLC showed a similar benefit for patients > 65 years old compared to others [17]. For SCLC, in both CASPIAN and IMpower-133, nearly half of the patients were ≥65 years old, and a chemoimmunotherapy combo had similar efficacy in these patients compared to younger patients [7,8]. However, the patients in these trials were highly selected, and the 65-year-old cut-off may not realistically reflect elderly population. We chose to study the efficacy of chemoimmunotherapy combo in unselected patients ≥70 years old and showed no evidence of benefit of this strategy compared to chemotherapy alone within the limits of a small sample size in these population sub-groups. Specific prospective studies in this population are needed to confirm these results.

Regarding performance status, some studies suggest a benefit of immunotherapy in patients with an impaired state (PS > 1) in contrast to the results obtained in our study. A monocentric retrospective single center in Israel (*n* = 104) showed a better OS with chemoimmunotherapy vs. chemotherapy (11.8 vs. 6.5 months) in SCLC patients [18]. Subgroups analyses showed a significantly better OS in case of PS 0–1, but no statistical difference for PS 2–3 [18].

Despite the high response rate in first-line treatment, tumor relapse occurs in the majority of the patients, with limited second-line treatment options. Considering the second-line treatment in advanced SCLC, topotecan, irinotecan or lurbinectedin are currently the only validated options in this setting [19,20]. However, their efficacy remains poor, with a median PFS of 4.2 months in randomized clinicals trials [19,20] and 1.9 months in real life [21]. In our study, we were able to describe the efficacy of these molecules after first-line treatment with IO-CT. Our data suggest a similar efficacy profile compared to second-line treatments, whether they are given after IO-CT or CT alone [22].

Our study has several limitations. It is an observational, retrospective study. There was neither randomization nor pairing between the two patient cohorts. However, we insured the comparability of the two groups by excluding patients with classical contra-indications for IO (steroids use or auto-immune disease). The clinical characteristics of the patients were similar between the two cohorts. Moreover, the timing of the tumor evaluation may have varied according to the centers, which may have potentially slightly changed between the two time periods selected in our study. This could partially explain the absence of difference in terms of PFS between the two cohorts in our study. Furthermore, the staging work-up and the treatment strategy may have varied in these different time periods. In addition, the lack of centralized image review can also limit the interpretation of the tumor response and PFS in our study. However, we were able to provide concordant data on the OS benefit with the association of atezolizumab and chemotherapy. The main strengths of our study were its multicenter design, as well as the inclusion of elderly patients and patients with impaired general condition and brain metastases who were excluded from clinical trials. The main results obtained corroborate those of the major international multicentric phase III trials that evaluated atezolizumab and durvalumab in the first-line treatment of metastatic SCLC.

The results of our real-life multicentric study validate those from the phase III IMpower 133 trial. Dedicated prospective trials should be conducted in elderly patients and those with poor PS to evaluate the efficacy of CT-IO in these populations. Further studies are also needed to better individualize patient subgroups who benefit the most from IO-CT in order to better personalize the treatment strategies in advanced SCLC. Recent studies are also looking at new therapeutic targets, in particular phase I/II studies looking into the use of anti-DLL3 antibodies, the results of which could prove promising. The search for new molecular signatures is likely to pave the way for new anti-cancer therapies in this field.

## 5. Conclusions

In conclusion, the results of our multicentric study on the combination of chemo- and immunotherapy in real life validate those of the IMpower 133 phase III trial and support the use of CT and IO as standard first-line treatments for patients with metastatic SCLC. 

## Figures and Tables

**Table 1:** Baseline characteristics of patients.

Characteristics		Cohort 1 (*n* = 96)	Cohort 2 (*n* = 57)	*p*-Value
**Age, mean (standard deviation)**		64.9 (8.39)	67.1 (9.04)	0.13
**Performance status**	0	22 (23)	14 (25)	0.21
	1	51 (53)	37 (65)	-
	2	20 (21)	5 (8.8)	-
	3	3 (3.1)	1 (1.8)	-
**Sex**	Man	62 (65)	31 (54)	0.21
	Woman	34 (35)	26 (46)	-
**Smoking habit**	Never	2 (2.1)	2 (3.5)	0.80
	Past	31 (32)	19 (33)	-
	Current	63 (66)	36 (63)	-
**Brain metastasis**	No	62 (65)	43 (75)	0.16
	Yes	34 (35)	14 (25)	-
**Liver metastasis**	No	53 (55)	32 (56)	0.91
	Yes	43 (45)	25 (44)	
**Pleural involvement**	No	68 (71)	28 (49)	<0.01
	Yes	28 (29)	29 (51)	-
**Platinum salt**	Cisplatin	18 (19)	0 (0)	<0.001
	Carboplatin	67 (70)	57 (100)	-

Data are presented as *n* (%) unless otherwise specified.

**Table 2:** Objective response rate.

	Cohort 1 (*n* = 96)	Cohort 2 (*n* = 57)	*p*-Value
Complete response	6 (6.2)	6 (11)	0.04
Partial response	53 (55)	40 (70)	-
Stability	12 (12)	6 (11)	-
Progression	25 (26)	5 (8.8)	-

Data are presented as *n* (%).

**Table 3:** Adverse events.

	Grade	Cohort 1 (*n* = 94)	Cohort 2 (*n* = 55)	*p*-Value
Hepatic cytolysis	1	3 (3.2)	2 (3.5)	0.28
	2	7 (7.4)	1 (1.8)	-
	3	0 (0)	1 (1.8)	-
Infection		36 (38)	29 (51)	0.13
Renal failure	1	4 (4.2)	2 (3.5)	1.00
	2	5 (5.3)	3 (5.3)	-
	3	3 (3.2)	1 (1.8)	-
	4	1 (1.1)	0 (0)	-
Pneumonitis		0 (0)	3 (5.3)	0.15
Skin toxicity	1	8 (8.4)	7 (12)	0.79
	2	5 (5.3)	2 (3.5)	-
	3	1 (1.1)	1 (1.8)	-
Gastrointestinal toxicity	1	21 (22)	9 (16)	0.02
	2	4 (4.2)	9 (16)	-
	3	9 (9.5)	1 (1.8)	-
	4	2 (2.1)	0 (0)	-
Hematological disorders	1	20 (21)	19 (33)	0.34
	2	16 (17)	10 (18)	-
	3	22 (23)	8 (14)	-
	4	19 (20)	8 (14)	-
Other toxicity		3 (3.2)	10 (18) *	<0.01

Data are presented as *n* (%) unless otherwise specified. * thyroiditis (*n* = 4), pancreatitis (*n* = 2), adrenal insufficiency (*n* = 1), diabetes (*n* = 1), hypophysitis (*n* = 1).

## Data Availability

The data presented in this study are available upon request from the corresponding author.

## Supplementary Materials

The following supporting information can be downloaded at: https://www.mdpi.com/article/10.3390/cancers15184593/s1. Supplementary Figure S1: Progression-free survival with 2nd line treatment in the 2 cohorts. Supplementary Figure S2: Progression-free survival with 2nd line treatment according to the type of treatment in cohort 2 (chemo-immunotherapy). Supplementary Figure S3: Overall survival of patients without (A) or with (B) brain metastases. Supplementary Figure S4: Overall survival of patients with (A) or without (B) liver metastases. Supplementary Figure S5: Overall survival in PS 0–1 (A) and PS ≥ 2 (B) patients. Supplementary Figure S6: Overall survival of patients < 70 yo (A) and ≥70yo (B).
