# Supplementary material for: Evaluation of Real-Life Chemoimmunotherapy Combination in Patients with Metastatic Small Cell Lung Carcinoma (SCLC): A Multicentric Case–Control Study
Source: Cancers (Basel). 2023 Sep 15;15(18):4593. doi: 10.3390/cancers15184593 (PMC10526821; doi:10.3390/cancers15184593)

Supplementary data

Supplementary Figure S1: Progression-free survival with 2nd line treatment in the 2 cohorts

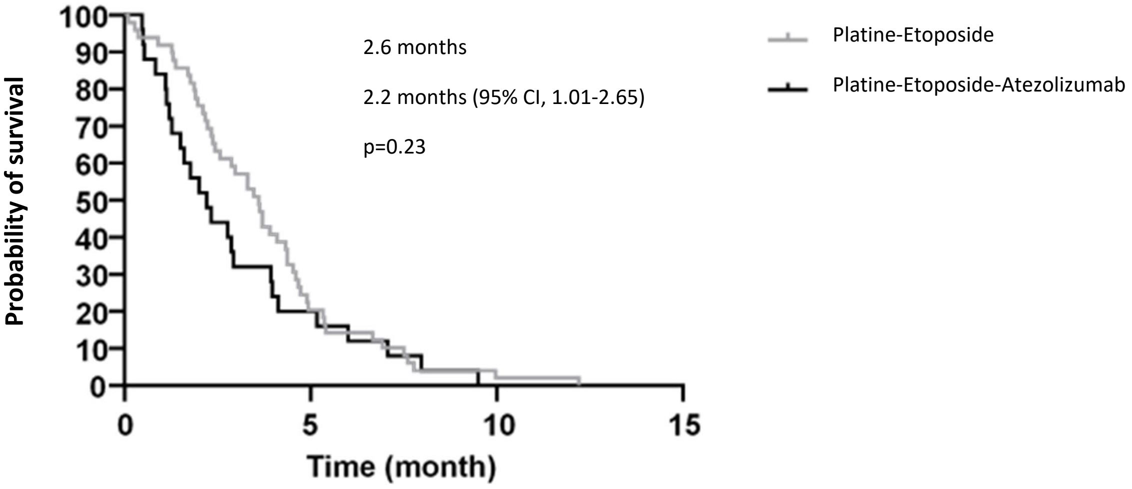

Cohort 1	49	11	2
Cohort 2	25	6	1

Supplementary Figure S2: Progression-free survival with 2nd line treatment according to the type of treatment in cohort 2 (chemo-immunotherapy)

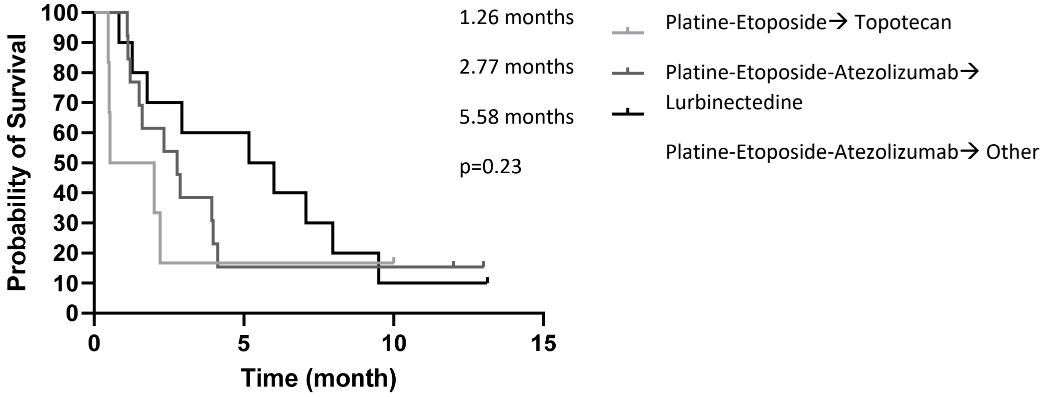

Topotecan	6	2	1
Lurbinectedine	13	1	1
Other	10	7	2

<u>Supplementary Figure S3:</u> Overall survival of patients without (A) or with (B) brain metastases

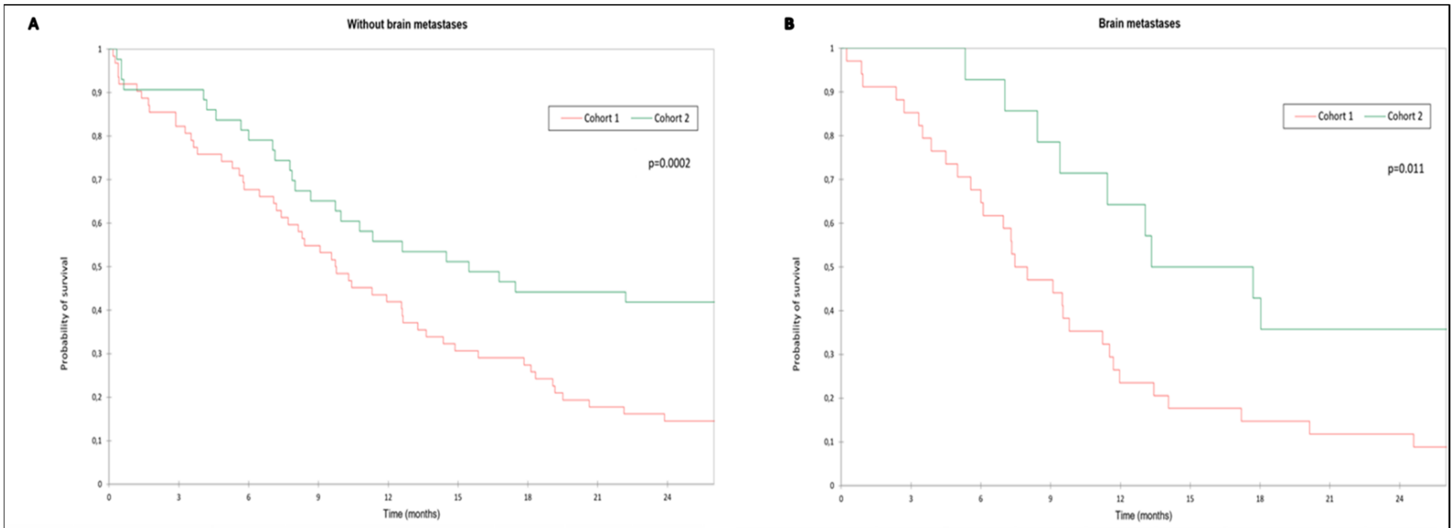

Cohort 1	63	53	43	35	27	20	18	12	11
Cohort 2	43	40	35	29	27	23	20	20	19

Cohort 1	34	30	23	17	9	7	6	5	5
Cohort 2	14	14	14	12	10	8	7	6	6

Supplementary Figure S4: Overall survival of patients with (A) or without (B) liver metastases

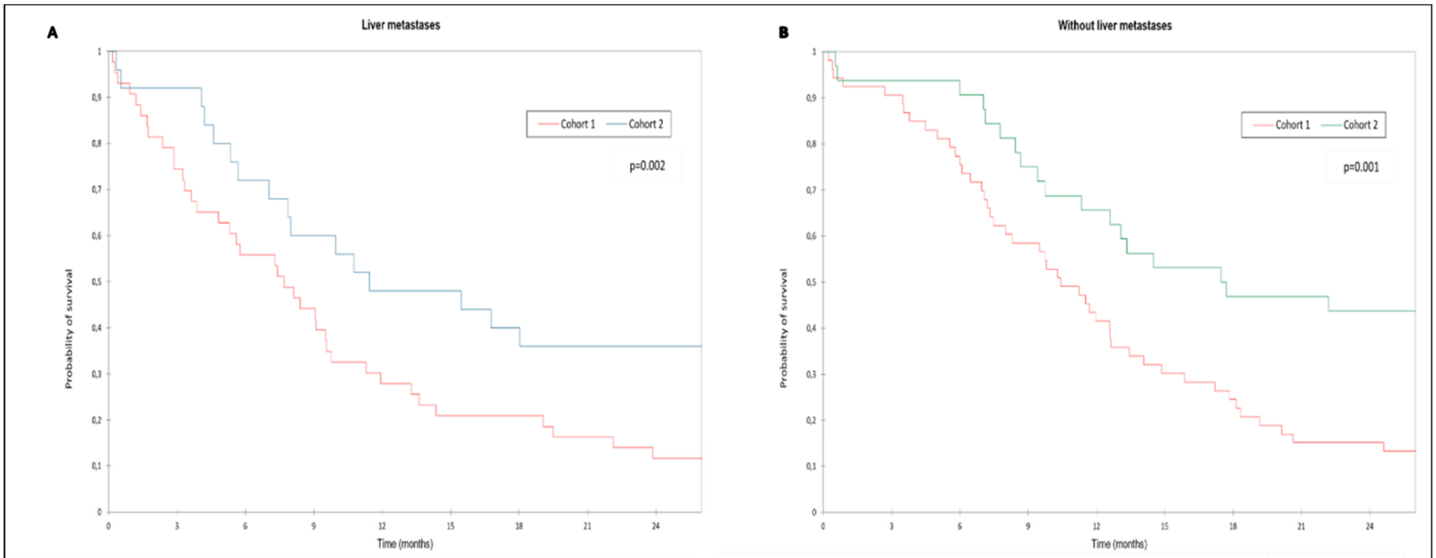

Cohort 1	43	34	25	20	13	10	10	8	6
Cohort 2	25	24	19	16	13	13	11	10	10

Cohort 1	53	50	41	32	23	17	14	9	9
Cohort 2	32	31	30	25	22	18	16	16	15

Supplementary Figure S5: Overall survival in PS 0-1 (A) and PS ≥2 (B) patients

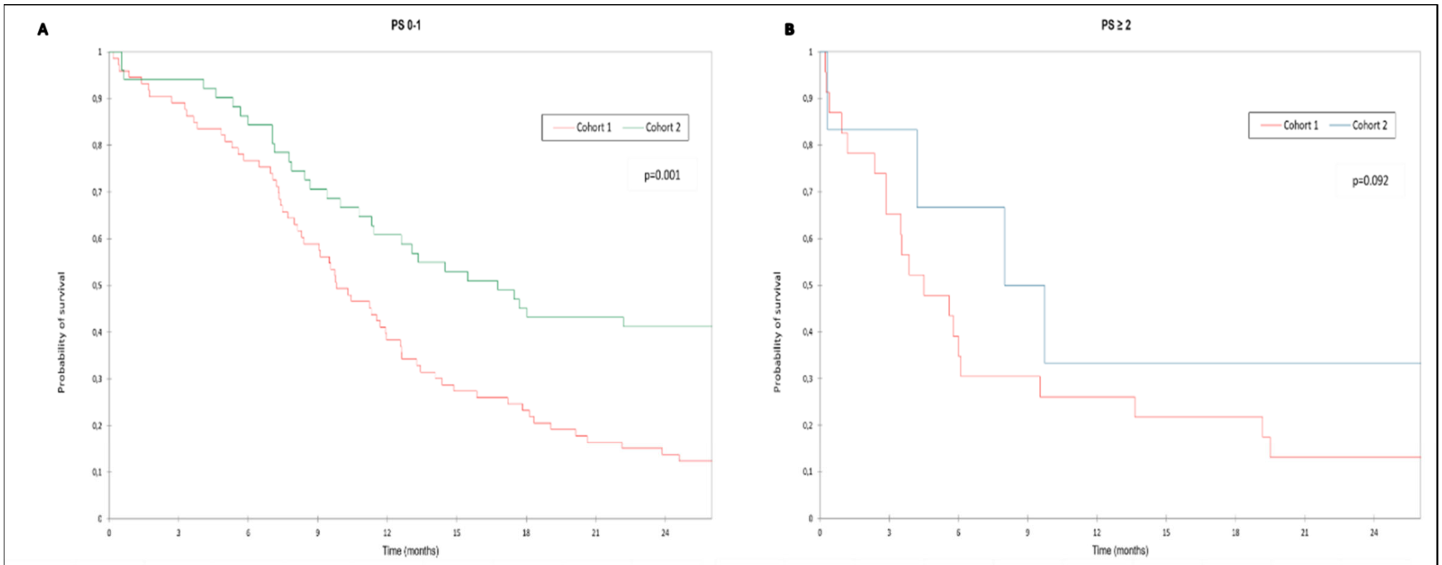

Cohort 1	73	66	57	44	29	21	18	13	11
Cohort 2	51	49	44	37	32	28	24	23	22

Cohort 1	23	17	9	8	7	6	6	4	4
Cohort 2	6	6	5	4	3	3	3	3	3

<u>Supplementary Figure S6</u>: Overall survival of patients <70 yo (A) and ≥70yo (B)

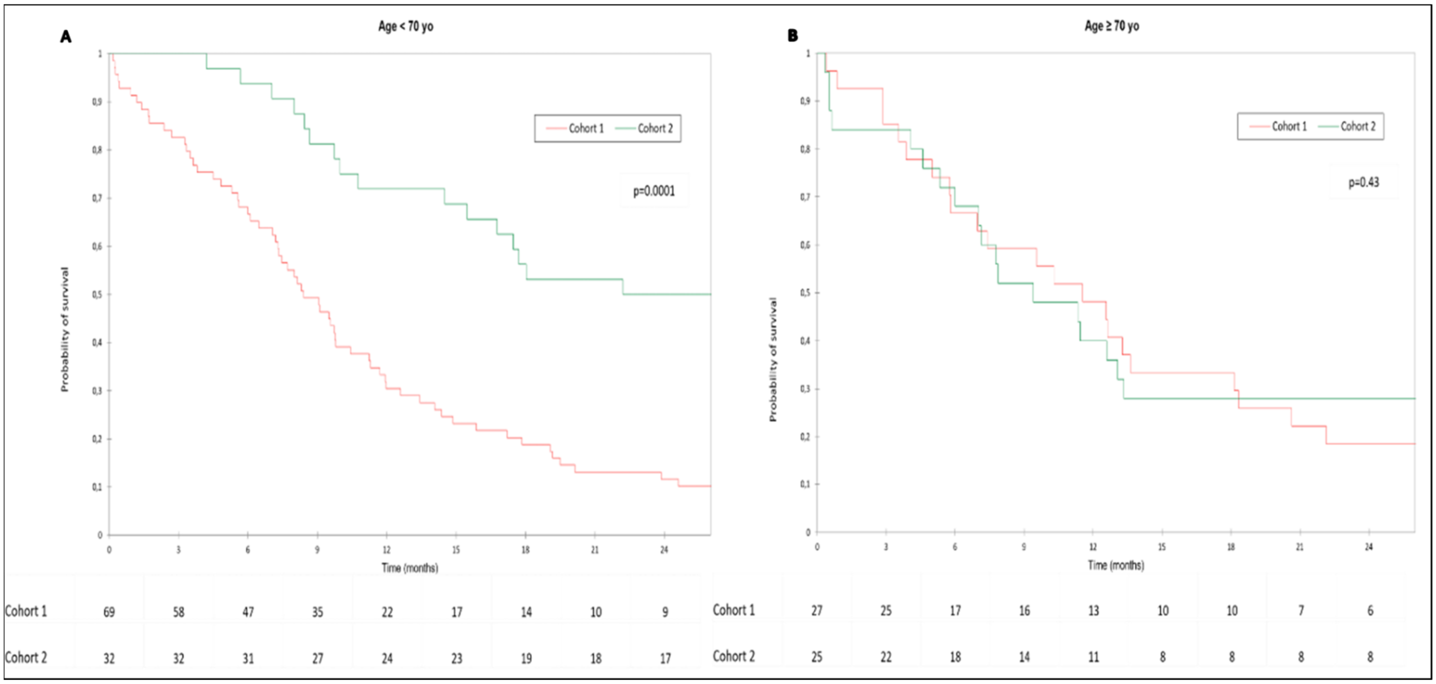

Supplement: Supplementary file 1 [file cancers-15-04593-s001.zip › cancers-2511835-supplementary.pdf]
